# Enhancing physical activity in older adults receiving hospital based rehabilitation: a phase II feasibility study

**DOI:** 10.1186/1471-2318-12-26

**Published:** 2012-06-08

**Authors:** Catherine M Said, Meg E Morris, Michael Woodward, Leonid Churilov, Julie Bernhardt

**Affiliations:** 1 Physiotherapy Department, Austin Health, Heidelberg West, VIC, Australia; 2 Physiotherapy, Melbourne School of Health Sciences, The University of Melbourne, Parkville, VIC, Australia; 3 Aged Care Services, Austin Health, Heidelberg West, VIC, Australia; 4 Florey Neuroscience Institutes, Austin Campus, Heidelberg, VIC, Australia

**Keywords:** Mobility limitation, Rehabilitation, Exercise therapy, Hospitalization, Randomized controlled trial

## Abstract

**Background:**

Older adults receiving inpatient rehabilitation have low activity levels and poor mobility outcomes. Increased physical activity may improve mobility. The objective of this Phase II study was to evaluate the feasibility of a randomized controlled trial (RCT) of enhanced physical activity in older adults receiving rehabilitation.

**Methods:**

Patients admitted to aged care rehabilitation with reduced mobility were randomized to receive usual care or usual care plus additional physical activity, which was delivered by a physiotherapist or physiotherapy assistant. The feasibility and safety of the proposed RCT protocol was evaluated. The primary clinical outcome was mobility, which was assessed on hospital admission and discharge by an assessor blinded to group assignment. To determine the most appropriate measure of mobility, three measures were trialled; the Timed Up and Go, the Elderly Mobility Scale and the de Morton Mobility Index.

**Results:**

The protocol was feasible. Thirty-four percent of people admitted to the ward were recruited, with 47 participants randomised to a control (n = 25) or intervention group (n = 22). The rates of adverse events (death, falls and readmission to an acute service) did not differ between the groups. Usual care therapists remained blind to group allocation, with no change in usual practice. Physical activity targets were met on weekdays but not weekends and the intervention was acceptable to participants. The de Morton Mobility Index was the most appropriate measure of mobility.

**Conclusions:**

The proposed RCT of enhanced physical activity in older adults receiving rehabilitation was feasible. A larger multi-centre RCT to establish whether this intervention is cost effective and improves mobility is warranted.

**Trial registration:**

The trial was registered with the ANZTCR (ACTRN12608000427370).

## Background

Loss of mobility, which may include difficulty changing body position, transferring from one place to another, or walking, is a major reason older adults are admitted for hospital-based rehabilitation [1]. Evidence indicates that mobility outcomes are sub-optimal in older people following discharge from an aged care rehabilitation facility [2]. Fourteen percent of older people discharged from rehabilitation were unable to walk 10 m [2]. Of those who could walk 10 m, only 31% were independent on steps. Gait speed was also significantly reduced, with a median speed of 0.46 m/sec (inter-quartile range 0.32), compared to a mean speed range of 1.2–1.3 m/sec in healthy older adults [2]. Poor mobility has serious consequences for older adults. It is associated with the need for long term care, [3] falls [4], loss of functional independence and mortality [5]. It is therefore important to maximise recovery of mobility in this older ‘at risk’ population.

While it is acknowledged that bed rest and inactivity are detrimental for mobility and function [6], there are no clinical guidelines on the optimum activity levels for older adults undergoing rehabilitation to improve mobility. Studies show that physical activity levels, or the amount of time spent performing movements that require energy expenditure, are low in rehabilitation settings [7-9], particularly in the late afternoon, during the evenings and on weekends [10]. Various studies [11-13] and a systematic review [14] have investigated the impact of increased physical activity on older adults admitted to an acute facility. However the effect of increased physical activity on the mobility of older adults in subacute rehabilitation has not yet been examined. This is best tested in a randomized controlled trial (RCT).

Before embarking on a large, multicentre RCT, the feasibility of the protocol should be demonstrated, particularly when the intervention is complex [15]. It is important to establish whether enhanced physical activity can be safely delivered as intended to a frail population of older adults. A limitation [14] of studies in the acute population was that the measurement tools utilised were not sensitive to changes in mobility, therefore an appropriate measure of mobility should be identified. We also wanted to know whether provision of enhanced physical activity to an intervention group leads to contamination, resulting in changes in usual care activity levels in either the control or intervention groups.

The research questions addressed in this Phase II feasibility study were:

1. Is the proposed RCT protocol of enhanced physical activity training for older adults during rehabilitation feasible and safe?

2. What is the most appropriate measure of mobility for this population?

3. What sample size is required to determine whether enhanced physical activity improves mobility?

## Methods

### Design

The study was a single blinded, RCT with intention to treat analysis. Participants were recruited from two aged care rehabilitation wards within a tertiary hospital. Most admissions to the wards were from an acute hospital. The study was approved by the hospital ethics committee (Austin Health Project No 03223) and the trial was registered with the ANZTCR (ACTRN 1260800042730). Consent was obtained from the participant within 48 hours of admission. If the participant was unable to provide consent due to cognitive impairment (defined as a Mini Mental State Examination score less than 25/30) consent was obtained from the ‘Person Responsible’. Participants were randomised to receive a program of enhanced physical activity or usual rehabilitation care.

### Participants

Participants were eligible for inclusion if they were aged over 60 and had ‘improve mobility/walking’ as a goal at admission. Participants were excluded if the primary reason for admission was to await residential care placement, they did not require physiotherapy or if there were medical restrictions on mobilisation (e.g. non weight bearing). As we did not have funding for interpreters to assist with the trial, people who did not speak English could only be recruited if next of kin were available to assist with consent.

### Randomisation

A blocked stratified randomization procedure, based on functional level, was used to allocate participants to either the intervention group, (enhanced physical activity), or a control group (usual care) following baseline assessment. This approach was taken to increase the chance that the groups had similar numbers of low and high functioning participants, and to ensure the intervention protocol was trialled with both low and high functioning participants. Functional stratification was according to two categories based on functional level; nonambulant and ambulant. Nonambulant participants were those unable to walk or requiring assistance of two people to walk at baseline assessment (functional levels 1 and 2, Table 1). Ambulant participants included those able to walk with assistance of one person, with supervision only or independently (functional levels 3 and 4, Table 1). Randomization was computer generated and performed by a third party. Allocation was concealed in opaque envelopes.

**Table 1:** Functional classification of participants and recommended activities for intervention group

**Level**	**Function**	**Intervention**
1	Patient is unable to transfer out of bed without maximum assistance (two persons or a hoist) and has poor static and dynamic sitting balance (unable to sit independently).	Bed exercise program (including lower limb, upper limb and abdominal strength and bed mobility) and sitting balance exercises.
2	Patient can transfer out of bed with assistance from one person, has independent sitting balance, but is unable to stand independently. Requires moderate assistance from two people to walk.	Sitting exercise program including targeted lower limb strengthening exercises. Sit to stand exercises, standing balance exercises, stepping / marching on the spot as able (using rails/ gait aids for safety as indicated). Activities from the previous level may be included if specifically indicated. For example, if the participant is unable to perform full range movement against the effects of gravity, specific lower limb muscle strengthening exercises may be performed on the bed.
3	Patient can walk with minimal assistance of one person.	Walking exercises, sit to stand exercises, standing balance exercises, and step up exercises. Targeted lower limb strength exercises (where possible closed chain or functional strengthening exercises).
4	Supervision only or independence with ambulation. Requires minimal assistance or supervision on stairs.	Stairs exercises, walking exercises (including outdoor mobility), step up exercises, standing balance exercises. Targeted lower limb strength exercises as indicated (where possible closed chain or functional strengthening exercises).

### Intervention

Both groups received usual care, which included therapy provided by a multidisciplinary team. All participants routinely received one to two sessions of physiotherapy from Monday to Friday. These sessions were either individual sessions supervised by a physiotherapist/physiotherapy assistant or group exercise classes designed to improve lower limb strength or balance, depending on participants’ functional status and goals.

Participants in the intervention group received an additional program of enhanced physical activity. This program focused on increasing the time participants spent performing mobility activities in the late afternoons/evening and on weekends, as activity levels at these times have been shown to be low [10]. The aim was to double the previously reported [10] time spent performing standing and walking activities in the late afternoon and evening on weekdays. On weekends, the aim was to increase time spent performing standing and walking activities so activity levels were the same as activity levels on weekdays (with usual care) [10]. The intervention was individually tailored for each patient according to functional level, as detailed in Table 1, and delivered by a physiotherapist or physiotherapy assistant. Progress was monitored in each session and the intervention was modified as the patient’s function improved. Functional levels utilised by Jones et al., [13] were modified to simplify patient classification and increase the emphasis on mobility. Time spent performing each activity (eg walking, standing, bed exercises) was recorded in 5 minute increments on a paper recording sheet by the therapist (available from the author by request), and reasons for non-delivery of an intervention session were recorded.

### Blinding and contamination

Assessments were performed by an assessor blinded to group allocation. Staff providing the intervention could not be blinded. There was a risk of contamination if usual care staff became aware of group assignment. To minimise changes in staff practice, clinical staff not directly involved in the study were not told the specific purpose of the study. Intervention staff were not involved in other aspects of the client’s care. To test whether usual care staff remained blind to treatment group, staff were asked to guess group allocation once a participant was discharged.

### Outcome measures

Outcomes were assessed at three time points. Baseline assessment was completed within 48 hours of admission. The second assessment was completed less than 48 hours prior to discharge. The final assessment was completed by mail out and phone 3 months following discharge. All data were collected by an assessor blinded to group assignment.

The primary outcome of interest was change in mobility from admission to discharge. Three potential measures of mobility were trialled, to determine the most appropriate measure for the larger RCT. These were the Elderly Mobility Scale (EMS) [16-18], the Timed Up and Go Test (TUG), [19,20] and the de Morton Mobility Index (DEMMI) [21]. The (EMS) is a valid and reliable [16,17] method of assessing and detecting changes in mobility in older adults [18]. Performance on tasks such as getting in and out of bed, standing from a chair and walking is observed and scored. The TUG provides a timed measure of mobility as participants stand up, walk 3 m, turn around and sit down. The DEMMI is scored using a 100 point Rasch analysed scale. Performance of a wide range of mobility tasks such as getting in and out of bed, standing from a chair and walking is observed and scored. The DEMMI has demonstrated reliability and validity in older adults [21].

A number of secondary outcome measures were also obtained, including subacute length of stay (LOS), function as measured by the Barthel Index [22] and discharge destination. Subacute length of stay (LOS) was calculated from rehabilitation admission to discharge and measured in days. If discharge was to a residential care facility, discharge date was considered to be the day on which residential care paperwork was completed. The Barthel Index is a valid [23,24] and reliable global measure of function. It was obtained on admission and discharge, and proxy Barthel scores were obtained at 3 months via phone interview [25,26].

Additional data collected at baseline in order to adequately describe the population included age, gender, admission diagnoses, acute hospital LOS, cognition assessed using the Mini Mental State Examination (MMSE) [27] and comorbidities, assessed using the Charlson comorbidity score [28].

### Adverse events

To monitor intervention safety, adverse events such as death, falls during the hospital stay and readmission to an acute service (during the hospital stay) were recorded.

### Analysis

We used a sample of convenience for this study, estimating that 50 participants would be sufficient to examine feasibility issues. To determine whether the desired increases in activity were feasible, time spent performing standing and walking activities during intervention sessions as a proportion of the intervention target time were calculated. To examine the utility of the proposed measures of mobility in this population, the number of people able to complete the EMS, TUG and DEMMI were calculated, and data were examined for floor and ceiling effects. To examine whether usual clinical staff remained blind to group assignment we used the kappa statistic to determine the agreement between the group to which staff believed a participant had been allocated and actual group allocation. To evaluate whether the study protocol impacted on usual care (contamination), usual care activity levels between groups were compared using a Mann–Whitney *U* test.

Secondary outcomes were assessed via inspection of descriptive data. Data on falls, readmission rates and mortality were inspected. Mortality at 3 months was compared between the groups by calculating the Risk difference.

We planned sample size calculations for the larger trial assuming two groups of equal size, a two tailed significance threshold alpha of 0.05 and power to yield a statistically significant result of 80% using the most appropriate measure of mobility.

## Results

### Participants

**Figure 1:**
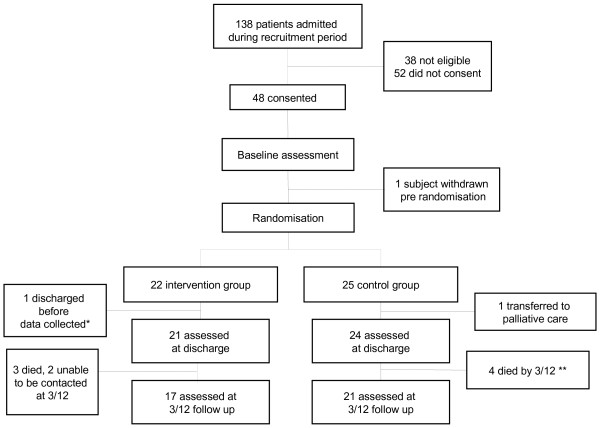
Participant flow through study.

Participant flow through the trial is illustrated in Figure 1. Baseline characteristics for both groups were similar, as shown in Table 2.

**Table 2:** Baseline characteristics

	**Control (n = 25)**	**Intervention (n = 22)**
Age	81.6 (sd = 6.5)	80.8 (sd = 4.6)
Gender (male)	15 (60%)	9 (41%)
Acute LOS (median days)	12(IQR 8.5–18)	15(IQR 11.5–20)
Admission Diagnosis		
Musculoskeletal^*^	10	4
Cardiac/Respiratory^*^	4	10
Other Surgical	5	1
Neurological	1	2
Falls/Functional decline	3	4
Other	2	1
MMSE ^†^ (median)(maximum score 30)	26(IQR = 24–28)	26.5(IQR = 25–27)
Charlson Comorbidity Index	2 (IQR = 1–3)	2 (IQR = 1–3)
DEMMI (mean)(maximum score 100)	43.2(sd = 16.2,Range 15–74)	41.3(sd = 12.9, Range 15–67)
Able to complete TUG (yes)	12 (48%)	11 (50%)
TUG score ^‡^ (mean, sec)	31.3 (sd =12.36)	35.5 (sd =11.8)
EMS score (median)(maximum score 20)	12(IQR = 7–17, Range 0–19	15(IQR = 7–17, Range = 0–18)
Barthel (median)(maximum score 100)	68(IQR = 60–78, Range 26–87)	66(IQR = 55–76, Range 9–87)

*LOS* Length of stay, *MMSE* Mini mental state examination, *DEMMI* de Morton Mobility Index, *TUG* Timed up and Go, *EMS* Elderly Mobility Scale.

^*^ Includes relevant surgical admissions.

† Two subjects in each group were unable to complete the MMSE as no interpreter was available.

‡ TUG score for participants able to complete task, Control *n* = 12, Intervention *n* = 11.

### Intervention delivery

Ninety percent of intervention sessions were delivered; 5.5% of sessions were not delivered due to participant refusal and remaining sessions were not delivered as the participant was unwell or unavailable. Examination of activity delivered in the intervention group demonstrated that the median activity achieved on weekdays was 87.3% of the target. On weekends, the median activity achieved was only 61.0% of the target.

### Blinding & contamination

Clinical staff correctly identified group assignment only 48% of the time (Kappa = .037, 95% CI = −.147; .221, *p* = .695). Given that there was a 50% chance of guessing the correct group, even without knowledge of group assignment, these results indicate staff were not able to identify to which group participants were assigned.

There was no difference in the time spent performing standing and walking activities in ‘usual care’ physiotherapy between the intervention group (12.4 minutes per day, IQR = 11.0) and the control group (10.8 minutes per day, IQR *=* 10.1), (*U* = 263.5, p = .806).

### Measurement of mobility

(IQR = 64–98, Range = 17–100)Inspection of the baseline data in Table 2 demonstrates that nearly 50% of participants were unable to complete the TUG on admission, indicating a floor effect for this population. All subjects were able to complete both the EMS and the DEMMI. Admission scores for the EMS suggested a ceiling effect, with 5 participants scoring 18 or more (out of 20) on admission. This was confirmed by inspection of discharge data, with 5 of the 45 participants assessed at discharge scoring the maximum of 20. In contrast, the DEMMI showed no floor or ceiling effects, and appeared to be the strongest performing measure of mobility for this population.

### Secondary outcomes

Secondary outcomes are provided in Table 3. The Barthel showed a ceiling effect at the 3 month mark, with 9 participants scoring the maximum score of 100.

**Table 3:** Outcomes at discharge and three months post discharge

		**Control**	**Intervention**
		**(n = 25)**	**(n = 22)**
Discharge			
	Change in DEMMI (mean)	7.2 (9.2)	9.6 (8.8)
	Rehabilitation LOS (median days)	15(IQR 13.0–22.5, Range = 8–41)	16(IQR 11–27.5, Range = 8–49)
			
	Barthel (median)(maximum score 100)	86.5(IQR = 68–98, Range = 33–100)	85(IQR = 73–95, Range = 41–100)
	Discharge destination		
	Home alone	4	3
	Home with carer	12	12
	Low level care	4	4
	High level care	4	2
	Other	1	1
3/12 post discharge			
	Barthel (median)(maximum score 100)	96(IQR = 87–100, Range = 26–100)	93(IQR = 64–98, Range = 17–100)

Note. 3/12 = three months.

### Adverse events

Two participants experienced a noninjurious fall (1 control group, 1 intervention) during the hospital stay, giving falls rates of 2.1 falls/1000 bed days and 2.3 falls/1000 bed days respectively. No falls occurred during intervention delivery.

No participants were readmitted to the acute hospital during their rehabilitation stay. No participants died during their hospital stay, but one control group participant was transferred to palliative care. Mortality did not differ between the two groups with three participants in the intervention group (13.6%) and four participants in the control (16.0%) group were dead at the 3 month follow up (risk difference −0.01, 95% CI -0.23; 0.21; p > 0.999).

### Sample size estimation

We identified the DEMMI as the strongest performing measure of mobility in this population. As shown in Table 3, on discharge control group participants improved their mean DEMMI score by 7.2 (*sd =* 9.2*)* points, while intervention group participants improved their mean DEMMI score by 9.6 (*sd =* 8.8*)* points. Based on these results, assuming two groups of equal size and using a two tailed significance threshold alpha of 0.05 and power to yield a statistically significant result of 80%, and allowing for 20% dropout, a sample size of 266 participants would be required in each group.

## Discussion

We have shown the feasibility of a RCT of enhanced physical activity in older adults undergoing rehabilitation. The program of enhanced physical activity was delivered as intended on weekdays, and demonstrates that older people in rehabilitation can be more active than they are currently. Delivering the intervention after hours ensured participants had adequate time to rest between exercise sessions, and contributed to the high compliance rate. On weekends, while activity was increased, intervention targets were not met. One reason targets may not have been met was that participants were only provided with one therapy session per day on weekends. Providing a second therapy session on weekend days may give participants sufficient time to rest between sessions and allow the activity target to be achieved. Weekends provide an ideal opportunity to increase activity in older people undergoing rehabilitation. Typically, therapy is limited on weekends (for example, our facility provides three hours of a physiotherapist and a physiotherapy assistant on Saturday to cover 56 beds) so there is potential to significantly increase activity levels by providing therapy on weekends. However, the staffing costs associated with providing therapy on weekends are higher, so the cost effectiveness of this strategy must be investigated. Intervention staff were not provided with any feedback on activity levels, which may also have contributed to reduced compliance with weekend activity levels. Results highlight the importance of continued monitoring of intervention dosage in rehabilitation trials, and the need for feedback to intervention staff to ensure the desired intervention ‘dosage’ is delivered [29].

A high proportion of patients admitted to the wards were eligible for inclusion (72%), and a high proportion of those eligible were recruited (48%). Given that consent had to be obtained and the baseline assessment completed within 48 hours of admission, we considered this recruitment rate to be acceptable. Consent could not be obtained from 35 people as they were either non English speaking or cognitively impaired (and the next of kin could not be contacted within 48 hours of admission). These results suggest that recruitment to a larger trial could be achieved in a timely manner and the use of interpreters could improve recruitment of people from a non- English speaking background. The broad inclusion criteria no doubt contributed to the high proportion of people eligible for inclusion. Admission diagnoses and co-morbities (Charlson Co-morbidity Index) varied as did functional status (Barthel Index scores). Eighteen percent of participants had cognitive impairment, indicated by a MMSE less than 25/30. Stratifying by baseline functional status appeared effective and although the admission diagnoses varied, results demonstrate that the intervention can potentially be implemented in a wide range of participants. If effective, results would be generalisable to a high proportion of older adults admitted for rehabilitation.

The DEMMI was able to be administered to participants with both low and high levels of mobility, and did not demonstrate floor or ceiling effects. In contrast, the TUG demonstrated a floor effect and the EMS had a ceiling effect for this population. The DEMMI was easily administered, taking on average 10 minutes, and required minimal equipment. The interval nature of the Rasch analysed scale allows parametric statistical analysis. The clinometric properties of the DEMMI have been rigorously examined, and it has demonstrated reliability and validity in an older population [21]. Furthermore, studies have shown the DEMMI is responsive to clinical change and that it can be used across a range of clinical settings [21,30-32].

Even though control and intervention participants were cared for on the same wards, there was no evidence of a change in usual care. Usual care staff correctly identified group assignment less than half the time, (i.e. no better than chance), indicating they were not aware of group assignment, and activity levels in usual care physiotherapy sessions did not differ between groups. This is similar to findings of Bernhardt et al. [33] in an acute setting, and provides evidence that contamination will not be a confounder, however it will be important to continue to monitor contamination in future studies. The intervention was safe, with no increase in adverse events.

### Study limitations

As this was a pilot study, it was not powered to detect significant changes in mobility. The potential effectiveness of the intervention may also have been diluted as the desired ‘dosage’ of intervention was not achieved on weekends. In addition, we did not examine the longer term impact of the intervention on mobility, or the impact of the intervention on secondary outcomes such as length of stay, function, discharge destination, readmission rate post discharge and quality of life. These issues will be considered in future studies. Inspection of the baseline data suggests that there may have been some differences between the groups; the control group had a higher proportion of males, a lower acute LOS, a lower EMS and there were differences in admission diagnosis. These differences occurred during the randomization process by chance, and it would be anticipated that with a larger sample the groups would be comparable. If differences were observed with a larger sample, these variables would be entered as covariates in analysis. Use of the Barthel to measure function may be limited due to the ceiling effect, so other measures should be considered. Future studies will also examine the cost effectiveness of the intervention, to enable clinicians and health care managers to make informed decisions about resource allocation.

## Conclusion

Increasing physical activity is a safe, simple, non-invasive intervention, with potential to improve mobility outcomes. This study provides evidence that the proposed protocol for an RCT to determine the effectiveness of increased activity in older adults undergoing rehabilitation is feasible, and has shown that the DEMMI is an appropriate tool to measure mobility for this population. We plan to consider the longer term impact of the intervention and cost effectiveness in a larger trial.

## Abbreviations

DEMMI: de Morton mobility index; EMS: Elderly mobility scale; LOS: Length of stay; MMSE: Mini Mental State Examination; RCT: Randomized controlled trial; TUG: Timed up and go.

## Competing interests

The authors declare they have no competing interests.

## Authors’ contributions

CS conceived of the study, participated in design, oversaw data collection, contributed to data interpretation and drafted the manuscript. MM, JB and MW participated in study design and data interpretation and contributed to manuscript preparation. LC was responsible for statistical analysis and contributed to manuscript preparation. All authors read and approved the final manuscript.

## Pre-publication history

The pre-publication history for this paper can be accessed here:

http://www.biomedcentral.com/1471-2318/12/26/prepub
